# Ammonium glycyrrhizin counteracts liver injury caused by lipopolysaccharide/amoxicillin-clavulanate potassium

**DOI:** 10.18632/oncotarget.18291

**Published:** 2017-05-30

**Authors:** Zugong Yu, Feng Wu, Jing Tian, Xuewen Guo, Ran An, Yangyang Guo

**Affiliations:** ^1^ Laboratory of Veterinary Pharmacology and Toxicology, College of Veterinary Medicine, Nanjing Agricultural University, Nanjing, Jiangsu Province, 210095, China

**Keywords:** lipopolysaccharide, amoxicillin clavulanate potassium, chicken primary hepatocytes, compound ammonium glycyrrhizin, antioxidant

## Abstract

We treated isolated chicken primary hepatocytes with lipopolysaccharide/amoxicillin clavulanate potassium (LPS/AC) to model liver injury and investigate its underlying mechanisms. We also used this model to assess the cytoprotective effects of compound ammonium glycyrrhizin (CAG) *in vitro*. LPS/AC-induced injury decreased cell viability and increased the activity of serum aspartate transaminase and alanine transaminase. Levels of superoxide dismutase, glutathione, and glutathione peroxidase were lower than control, while levels of the oxidative product malondialdehyde and reactive oxygen species were higher. Treatment with CAG for 24 h ameliorated these changes. Caspase-3 activity assays and flow cytometry revealed increased apoptosis in the model group. However, apoptosis decreased after CAG treatment, as confirmed by Hoechst 33342 staining. We also observed changes in mitochondrial ultrastructure. Real-time PCR and western blot analyses showed that CAG treatment downregulated LPS/AC-induced RNA expression of caspase-3, caspase-9, bax, cytochrome c, and fas, and upregulated the expression of bcl-2. Mitochondrial cytochrome c was released into the cytosol and the inner mitochondrial membrane potential (ΔΨm) was decreased. Our results highlight CAG as a potential therapeutic agent to counteract LPS/AC-induced liver injury.

## INTRODUCTION

Lipopolysaccharide (LPS), a cell wall component of gram-negative bacteria [1], is capable of eliciting inflammatory responses that involve the release of numerous proinflammatory cytokines, thereby leading to hepatic necrosis and a decrease in the levels of antioxidant enzymes and free radical scavengers [2, 3]. In humans, the injection of nanograms of LPS into the bloodstream can result in septic shock [4], whereas its administration to animals induces symptoms of liver injury.

From 1995 to 2005, 77 out of 1,164 cases (6.6% incidence) in an outpatient hepatology clinic involved liver injury, which is commonly associated with the use of drugs [5]. Serious drug-induced liver injury may lead to hospitalization, and it is the most common identifiable cause of acute liver failure in the US [6, 7]. Antibacterial agents and other drugs are another frequent cause of liver failure after transplantation, autoimmune hepatitis, and drug-induced liver injury (DILI) [8–10]. It is reported that from 2004 to 2007, antibacterial agents including amoxicillin/clavulanic acid, third-generation cephalosporins, and fluoroquinolones, accounted for 45.5% of DILI in the US [11–13]. DILI arises via complex multi-step mechanisms, which are initiated by chemical insults to liver cells. Formation of chemically reactive metabolites, impairment of mitochondrial function, inhibition of the activity of the bile salt export pump (BSEP) and/or other biliary efflux transporters are some of the major processes that trigger DILI [14].

Amoxicillin/clavulanic acid is one of the most frequently used antibiotic combinations in both human and veterinary clinical practice [15]. Nonetheless, when amoxicillin is used alongside a β-lactamase inhibitor such as clavulanic acid, the risk of hepatotoxicity and liver injury is increased [16–18]. It is reported that amoxicillin clavulanate (AC) is a type of penicillin strongly associated with hepatotoxicity and is the most frequent cause of DILI-related hospitalizations in clinical medicine [19].

Gram-negative bacteria are common pathogens that attack chickens. Multiple antibiotics, particularly bactericidal drugs, have been used in high doses and frequencies in veterinary clinics [20]. LPS and AC can each lead to hepatic injury, and studies have shown that AC potassium induces the release of LPS. Glycyr1rhizic acid (GA) or glycyrrhizin is commonly used in Asia to treat patients with chronic hepatitis [21–23]. Compound ammonium glycyrrhizin (CAG), which is mainly composed of glycyrrhizin, glycine, and methionine, is an effective anti-inflammatory, anti-cancer, anti-hepatotoxic, and antioxidant drug [24–28].

Here, we used cultured chicken liver primary cells to investigate whether the release of LPS due to AC potassium administration aggravates hepatocyte injury. We created a chicken primary hepatocyte model to explore the mechanisms underlying liver damage by LPS/AC and to determine whether CAG imparts a protective effect. Using this model, we measured various anti-oxidative indicators such as superoxide dismutase (SOD), reduced glutathione (GSH), glutathione peroxidase (GSH-Px), reactive oxygen species (ROS), and malondialdehyde (MDA). We also measured alanine transaminase (ALT) and aspartate transaminase (AST) activity in liver cells, the percentage of apoptotic cells, and the expression levels of various mRNAs and proteins related to the apoptosis and p38 pathways.

## RESULTS

### Isolation and culture of chicken primary hepatocytes

**Figure 1:**
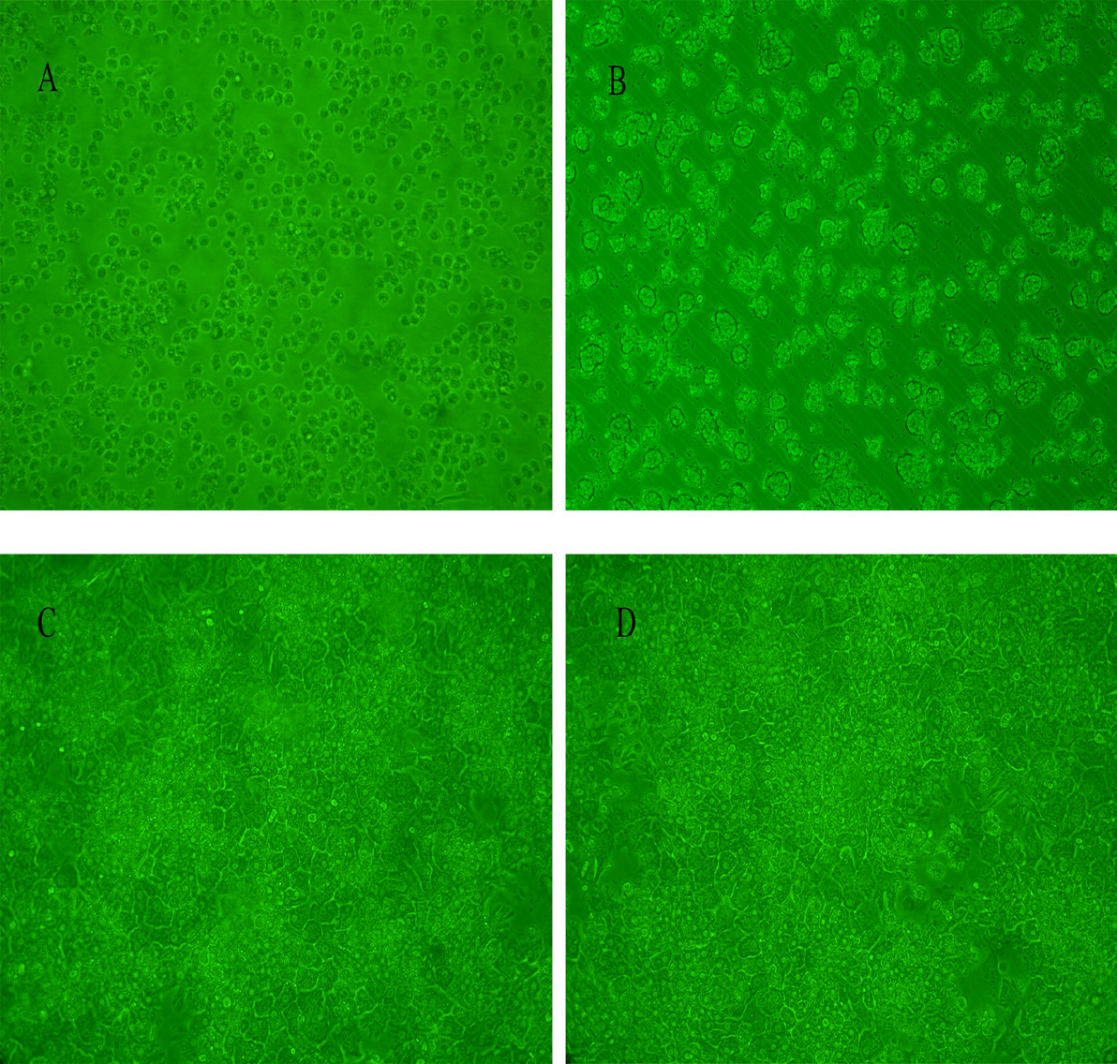
Cell viability assay for cultured chicken hepatocytes (**A**) Graph indicating that cell viability was ∼90%. (**B**) Image showing that most hepatocytes cultured for 6 h adhered to the bottom of the culture plate. (**C**–**D**) Hepatocytes cultured at 24 h and 48 h, exhibiting differentiation into irregular polygonal shapes.

Inverted phase contrast microscopy indicates that the hepatocytes that were isolated at an early stage were elliptical and circular in shape. Most of the hepatocytes adhered to the bottom of the culture plate at 6 h after plating. At 24 h of culture, the cells had fused and differentiated to form islands on the plate. At 9 days of culture, roughly half of the cells had undergone apoptosis, and the remaining cells showed cytoplasmic granulation (Figure 1).

### Effect of LPS/AC treatment on hepatocytes

**Figure 2:**
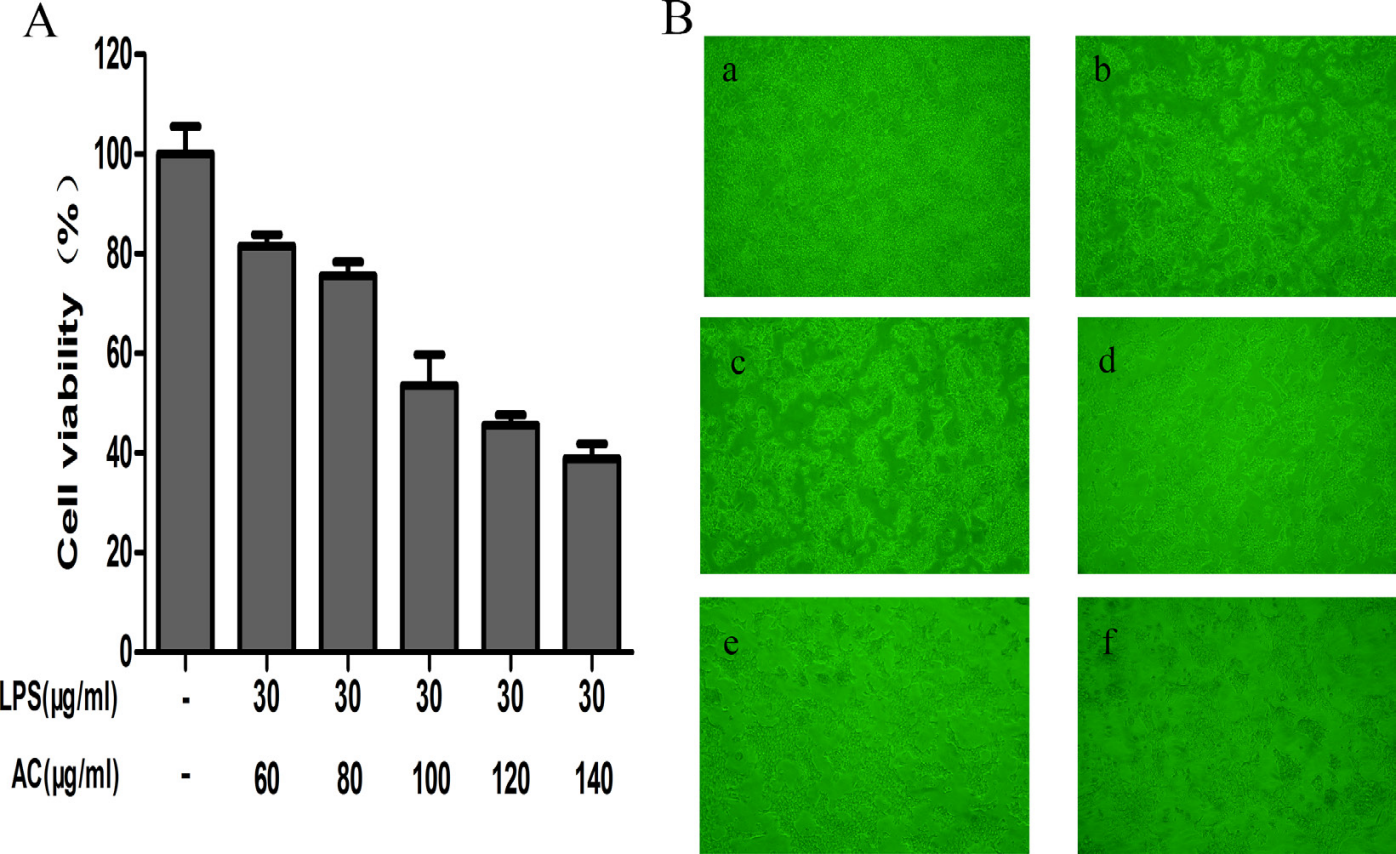
Effects of LPS/AC treatment on hepatocytes (**A**) Variations in hepatocyte viability after LPS/AC treatment for 24 h. MTT assay showing that LPS/AC treatment induces a decrease in cell viability in a dose-dependent manner. Values are expressed as the mean ± SD (*n =* 3) and the IC50 was 30 μg/mL of LPS + 100 μg/mL of AC. (**B**) Effect of different concentrations of LPS/AC treatment (24 h) on hepatocyte cytomorphology as visualized with an inverted phase-contrast microscope (20×). a: Normal cells; b: 30 μg/mL of LPS + 60 μg/mL of AC; c: 30 μg/mL of LPS + 80 μg/mL of AC; d: 30 μg/mL of LPS + 100 μg/mL of AC; e: 30 μg/mL of LPS + 120 μg/mL of AC; f: 30 μg/mL of LPS + 140 μg/mL of AC.

LPS/AC treatment of hepatocytes (Figure 2) resulted in variations in cell viability and morphology. Treatment of cells with 30 + 60 μg/mL and 30 + 80 μg/mL LPS/AC did not induce changes in cellular morphology, although their relative cell viability was 81.52 ± 2.35% and 75.54 ± 2.79%, respectively. At a concentration of 30 + 100 μg/mL, the observed cell viability of the hepatocytes was 53.56 ± 6.17% and their shape was irregular, with disruption of the cell membrane. Exposure of the cells to 30 μg/mL of LPS + 140 μg/mL of AC led to a great reduction in the total number of hepatocytes. The application of 30 μg/mL of LPS + 100 μg/mL of AC caused the death of 50% of the cells; therefore, this was deemed the most appropriate concentration in the model groups.

### CAG attenuates LPS/AC-induced acute liver injury in hepatocytes

**Figure 3:**
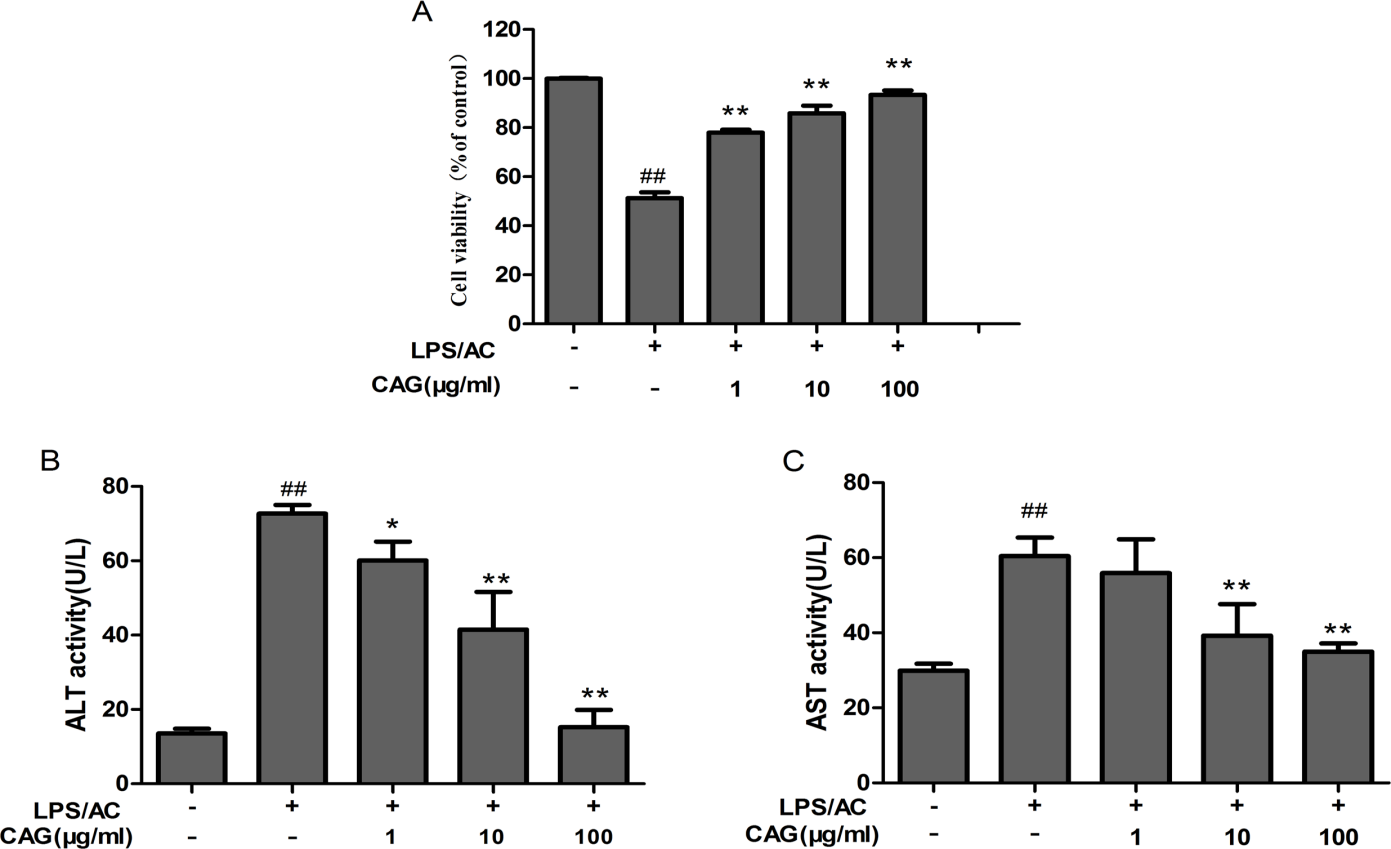
The effects of CAG on (**A**) cell viability, (**B**) alanine transaminase (ALT) activity, and (**C**) aspartate transaminase (AST) activity. The hepatocytes were treated with 30 μg/mL of LPS + 100 μg/mL of AC after exposure to CAG for 24 h. For the LPS/AC-treated groups, “−” and “+” represent the cells in culture medium and those treated with 30 μg/mL of LPS + 100 μg/mL of AC, respectively. For the CAG-treated groups, “−” represent cells treated without CAG. Values are expressed as the mean ± SD. (*n =* 3). ^#^ < 0.05, ^##^ < 0.01 compared to the control; * < 0.05, ** < 0.01 compared to the model.

The cells were divided into five groups: The control group neither received CAG nor LPS/AC; the model group was exposed to 30 μg/mL of LPS + 100 μg/mL of AC for 24 h; and the combination group was treated with 30 μg/mL of LPS + 100 μg/mL of AC for 24 h after the addition of 1, 10, or 100 μg/mL of CAG. Figure 3A shows that the activity of the cells was almost half of that of the control when exposed to 30 μg/mL of LPS + 100 μg/mL of AC for 24 h (51.23 ± 2.46%). However, cell viability reached 85.88 ± 3.03% and 93.37 ± 1.80%, respectively, when treated with 10 μg/mL and 100 μg/mL of CAG. The activity of both ALT and AST was significantly lower compared to that in the LPS/AC group (*P <* 0.01, Figure 3B–3C).

### Effect of CAG on the levels of SOD, GSH, GSH-Px, MDA, and ROS in hepatocytes

**Figure 4:**
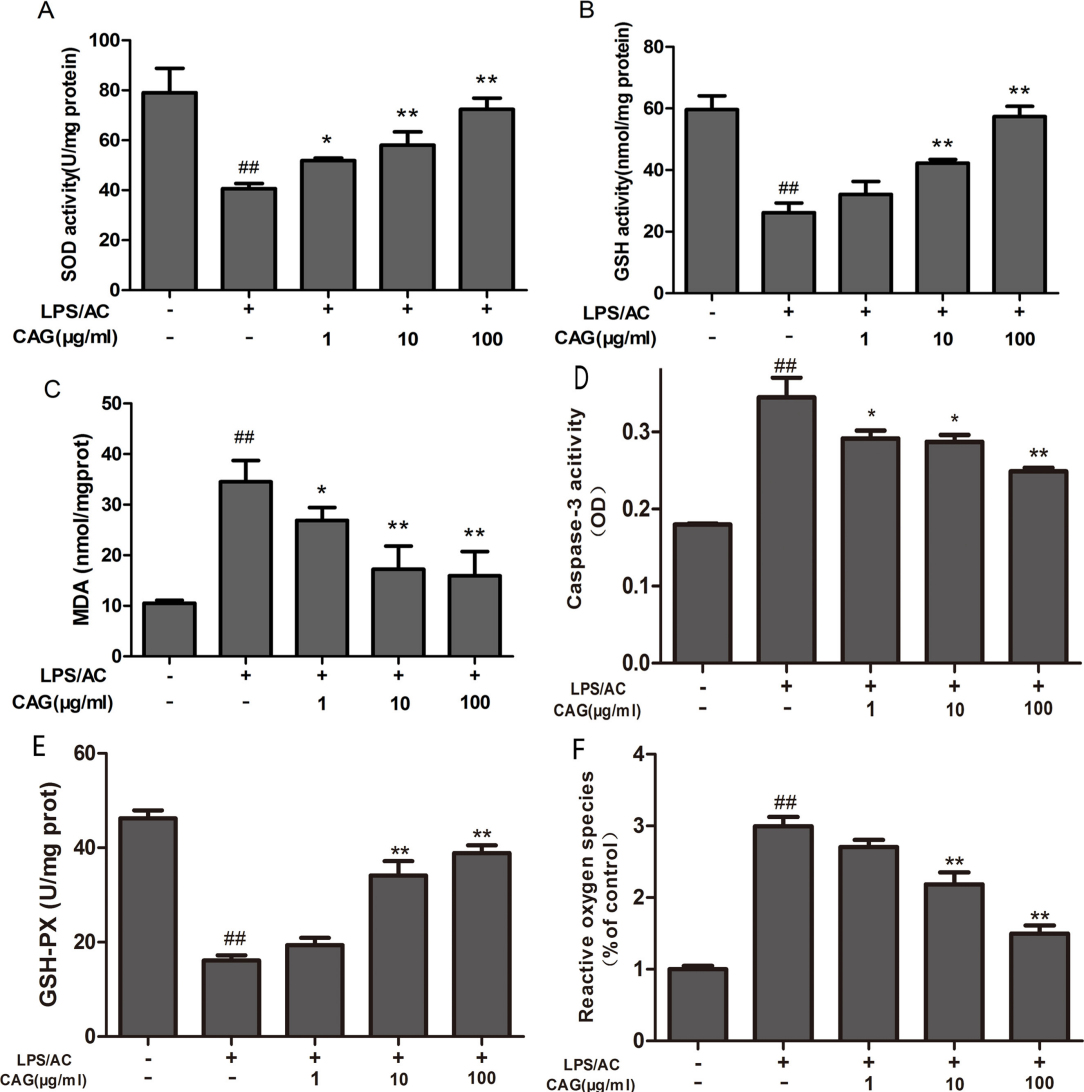
Effects of the CAG treatment on the levels of oxidative stress, cellular antioxidant enzymes, and caspase-3 activity (**A**) SOD, (**B**) GSH, (**C**) MDA, (**D**) caspase-3, and (**E**) GSH-Px activity, and (**F**) reactive oxygen species (ROS) levels in the supernatant. Values are expressed as the mean ± SD. (*n* = 3). ^#^ < 0.05, ^##^ < 0.01 compared to the control; * < 0.05, ** < 0.01 compared to the model.

Figure 4 shows that the administration of LPS/AC for 24 h led to a decrease (*P <* 0.01) in the activity of GSH, GSH-Px, and SOD to 26.18 ± 3.08 nmol/mg protein, 16.11 ± 0.30, and 40.63 ± 2.05 U/mg protein, respectively, compared to that in the control group (GSH: 59.64 ± 4.45 nmol/mg protein; GSH-Px: 46.22 ± 0.08 U/mg protein; and SOD: 79.05 ± 9.72 U/mg protein). Furthermore, treatment with LPS/AC resulted in an increase in MDA and ROS levels. Treatment with CAG (10, 100 μg/mL) for 24 h resulted in an increase in SOD, GSH, and GSH-Px activity (*P <* 0.05), and a decrease in MDA and ROS levels (*P <* 0.01). Together, these findings suggest that CAG treatment protects hepatocytes from LPS/AC-induced acute liver injury *in vitro*.

### Measurement of caspase-3 activity by using a colorimetric assay

The exposure of hepatocytes to LPS/AC for 24 h resulted in an increase in the activity of caspase-3 (OD: 0.344) compared to that in the control group (OD: 0.179) (*P <* 0.01). Moreover, the caspase-3 activity of hepatocytes exposed to CAG (1 and 10 μg/mL) (OD: 0.291, 0.287) significantly decreased (*P <* 0.05) compared to that in the model group that was exposed to a high concentration of CAG (100 mg/mL, *P <* 0.01, Figure 4D).

### Apoptosis rates of the LPS/AC and CAG groups as assessed by FCM

**Figure 5:**
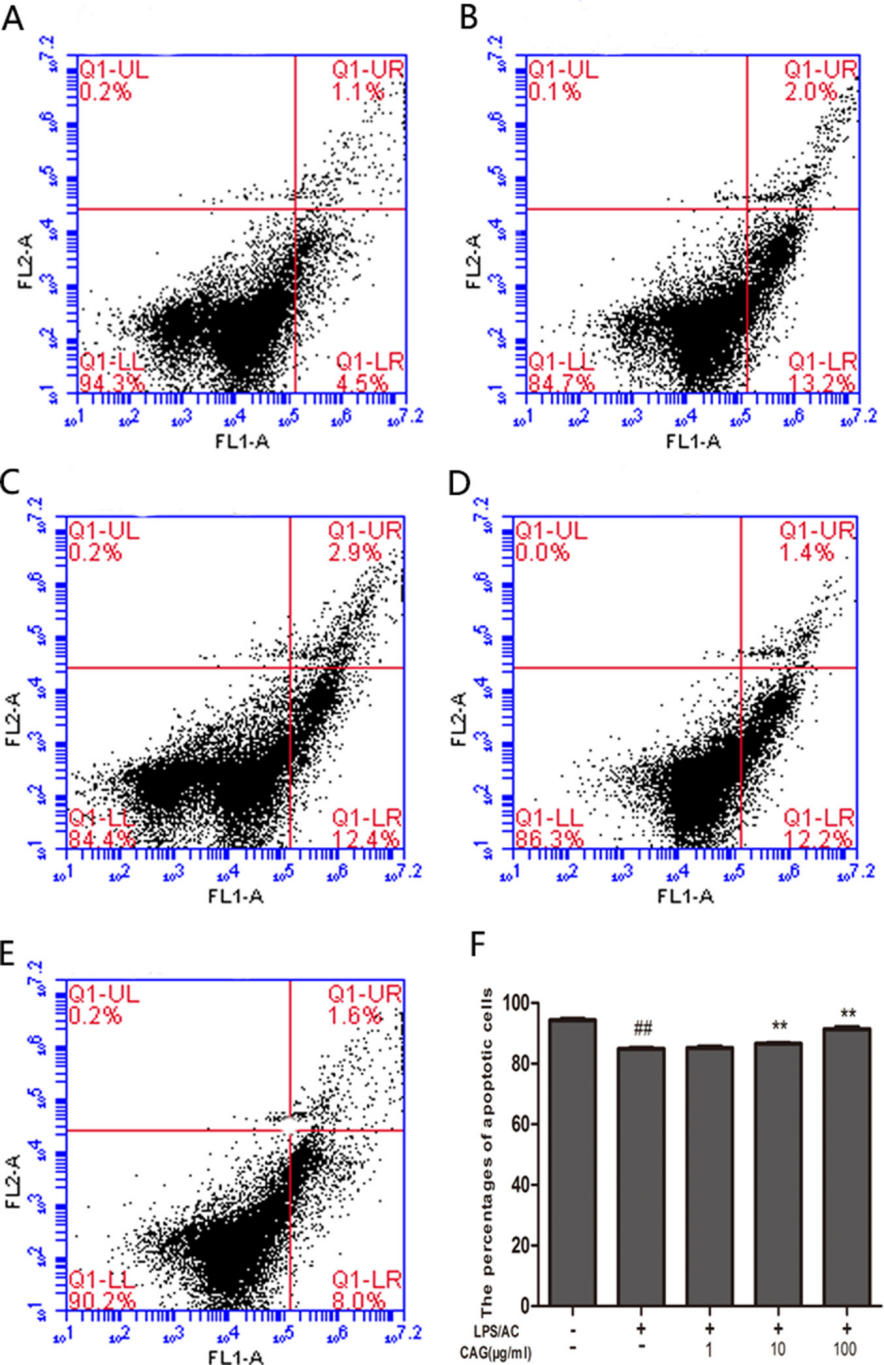
Changes in apoptosis rate in chicken primary hepatocytes as determined by FCM Change in the percentage of apoptotic cells for (**A**) control group, (**B**) model group, (**C**) 1 μg/mL CAG treatment group, (**D**) 10 μg/mL CAG treatment group, and (**E, F**) 100 μg/mL CAG treatment group. ^#^ < 0.05, ^##^ <0.01 compared to the control; * < 0.05, ** < 0.01 compared to the model group.

The percentage of apoptotic cells was higher in the LPS/AC group than in the control group (*P <* 0.05). On the other hand, the percentage of apoptotic cells in the groups exposed to 1, 10, or 100 μg/mL of CAG was lower than that in the model group (Figure 5).

### Assessment of apoptotic cells by Hoechst 33342 staining

**Figure 6:**
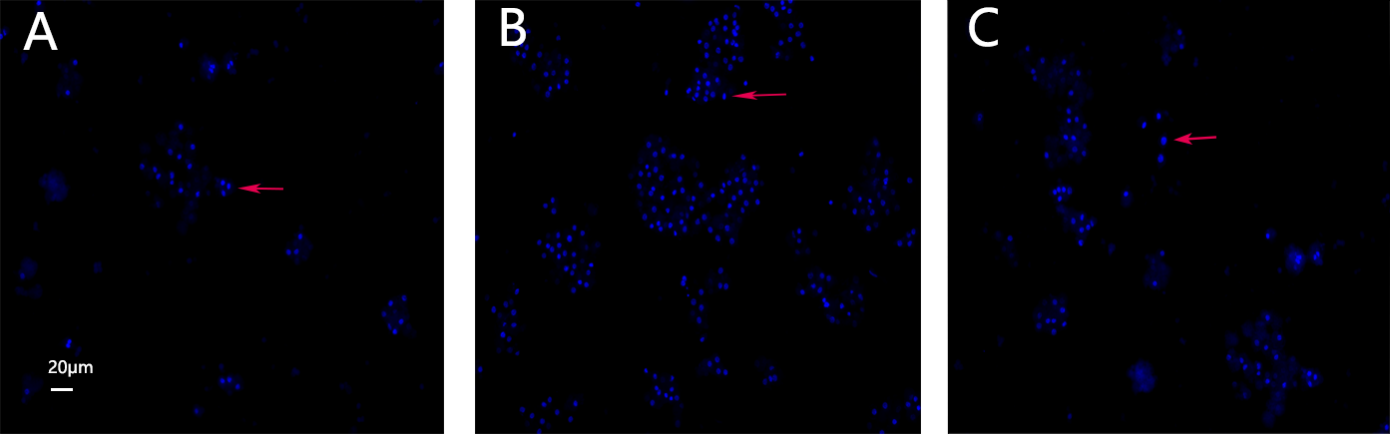
Hoechst 33342 staining Images of cultured hepatocytes stained with Hoechst 33342. (**A**) Control, (**B**) model, and (**C**) 100 μg/mL CAG treatment groups.

Compared to the control group, a more intense blue fluorescence due to Hoechst 33342 staining highlighting apoptotic cells was observed in the model group. However, after the exposure of the hepatocytes to 100 µg/mL CAG for 24 h, the rate of apoptosis decreased (Figure 6).

### CAG modifies the level of mRNA expression in hepatocytes

**Figure 7:**
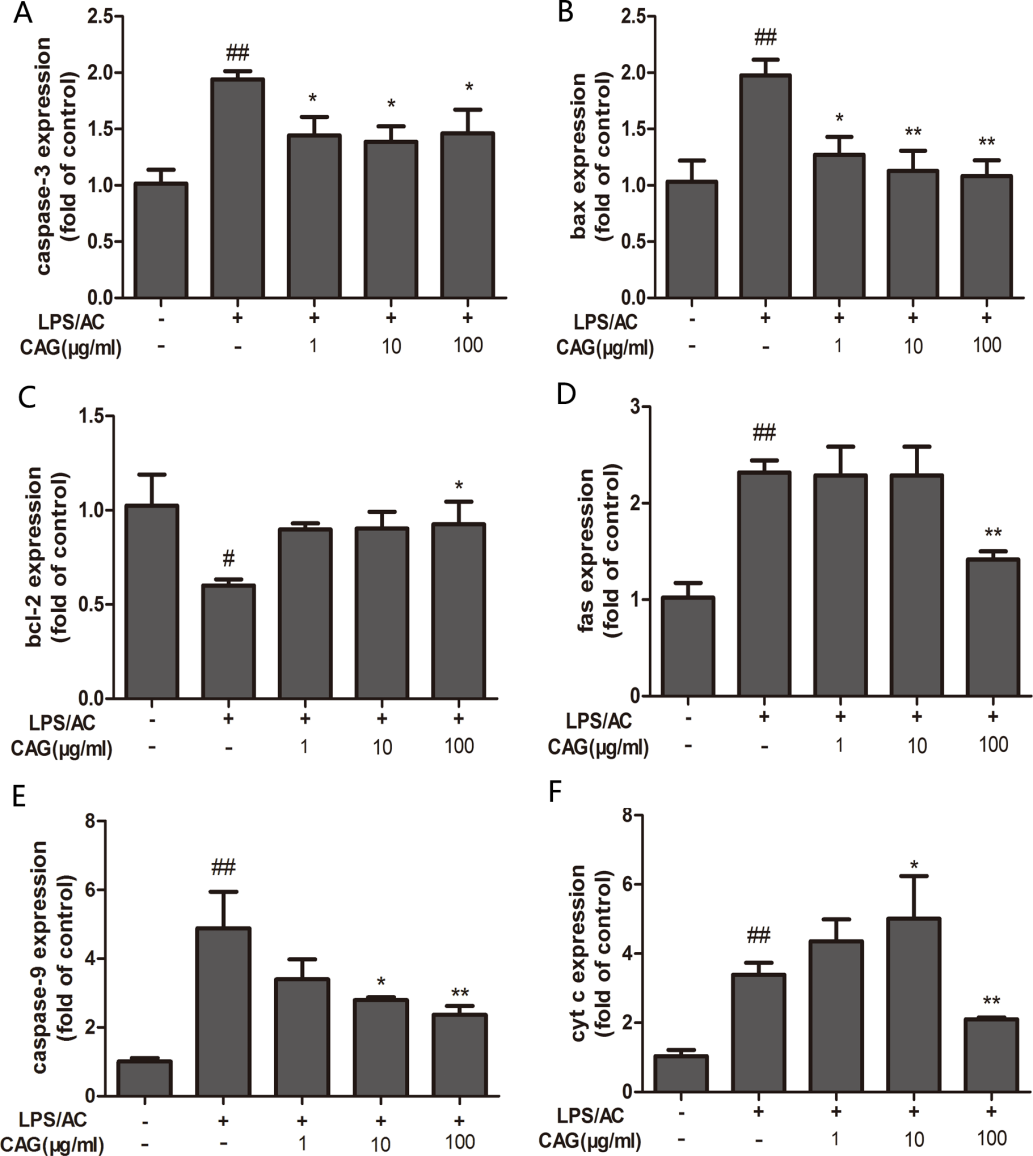
Effect of CAG on LPS/AC-induced changes in the expression of apoptosis-related genes in hepatocytes β-actin was used as a reference. mRNA expression of (**A**) caspase-3, (**B**) bax, (**C**) bcl-2, (**D**) fas, (**E**) caspase-9, and (**F**) cytochrome c. The 2^*–*ΔΔCt^ method was used to quantify the expression levels of each gene. Values are expressed as the mean ± SD (*n =* 3). ^#^ < 0.05, ^##^ < 0.01 compared to the control; * < 0.05, ** < 0.01 compared to the model.

At the transcriptional level, the application of 30 μg/mL of LPS + 100 μg/mL of AC triggered an increase in the expression of caspase-3, caspase-9, bax, Fas, and cyt c by 1.94 ± 0.13-fold, 4.88 ± 1.8-fold, 1.97 ± 0.24-fold, 2.32 ± 0.21-fold, and 5.01 ± 0.56, respectively, and a decrease in bcl-2 expression of 0.60 ± 0.05-fold in the hepatocytes, compared to levels in the control group. We did not observe differences in the levels of bcl-2 and fas mRNA expression between the model group and the groups treated with CAG at concentrations of 1 and 10 μg/mL. However, treatment with 100 μg/mL of CAG did result in decreased mRNA levels of bcl-2 and fas in hepatocytes compared to those in the model group (*P <* 0.05, Figure 7).

### Impact of CAG on LPS/AC-induced protein expression of caspase-3, caspase-9, bax, cyt c, and bcl-2

**Figure 8:**
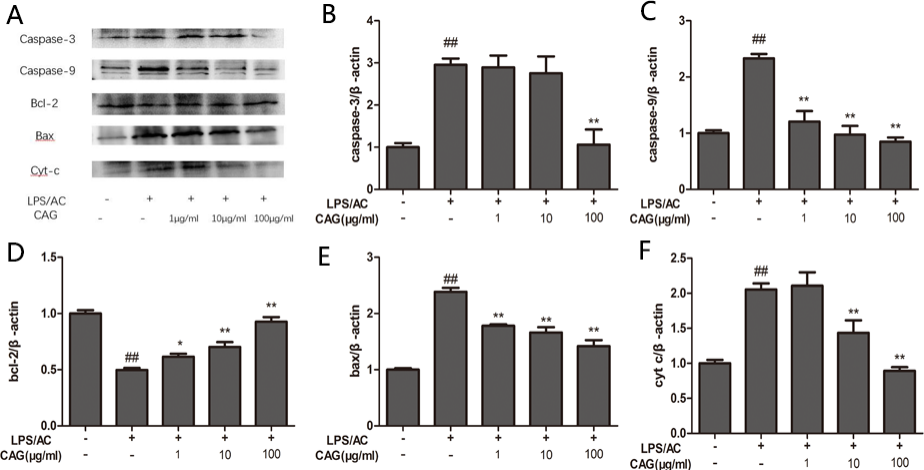
Effect of CAG on LPS/AC-induced changes in the expression of apoptosis-related proteins in hepatocytes (**A**) western blot bands, (**B**) caspase-3, (**C**) caspase-9, (**D**) bcl-2, (**E**) bax, and (**F**) cytochrome c. β-actin was used as reference. Bands analyzed by ImageJ. The data are expressed as the mean ± SD (*n =* 3). ^#^ < 0.05, ^##^ < 0.01 compared to the control; * < 0.05, ** < 0.01 compared to the model group.

The level of bax, caspase-3, and caspase-9 protein expression increased in the LPS/AC group compared to that in the control group and decreased in the presence of CAG relative to that in the model group (Figure 8). However, bcl-2 levels decreased in the LPS/AC group compared to those in the control group and increased in the CAG groups compared to those in the model group. The expression of cyt c was increased in the cytoplasm but decreased in mitochondria when cells were treated with LPS/AC. However, cyt c expression was decreased in the cytoplasm and increased in mitochondria compared with the model group when the concentration of CAG was 100 μg/mL (Figure 10).

**Figure 9:**
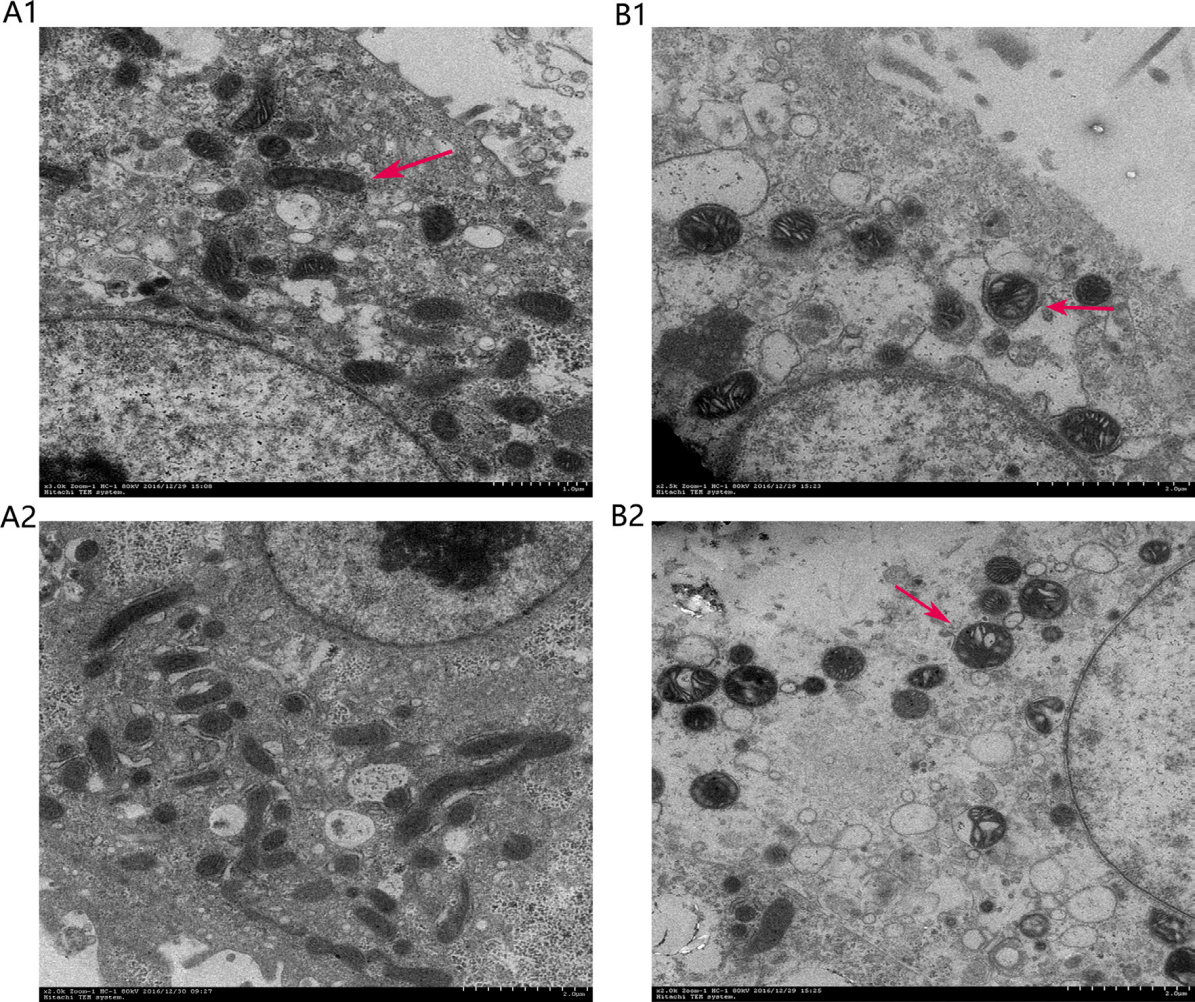
Ultrastructural features of chicken primary hepatocytes (**A**) Control and (**B**) model groups. Arrows point to mitochondria.

**Figure 10:**
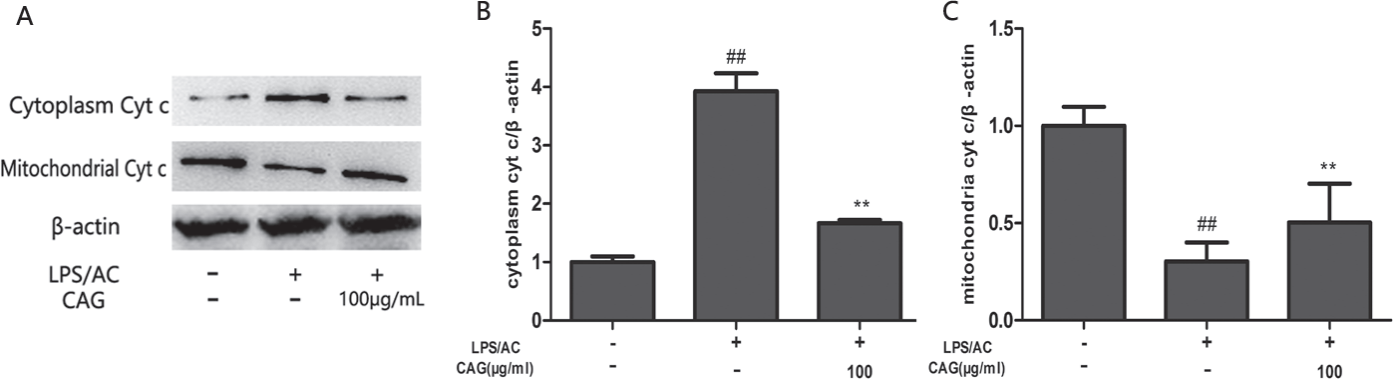
Effect of CAG on LPS/AC-induced changes in the expression of cytoplasmic and mitochondrial cyt c in hepatocytes (**A**) western blot bands, (**B**) cytoplasm cyt, and (**C**) mitochondria cyt c. β-actin was used as a reference. Bands analyzed by ImageJ. The data are expressed as the mean ± SD (*n =* 3). ^##^ < 0.01 compared to control group; ** < 0.01 compared to the model group.

### Ultrastructural changes in hepatocytes

The cells of the control group showed normal ultrastructural features, including a smooth round nucleus with the nuclear membrane intact, and mitochondria with normal cristae (Figure 9). The mitochondria of liver cells from the LPS/AC treatment group were swollen, vacuolated, and exhibited disintegration or loss of cristae.

### Effect of LPS/AC on mitochondrial membrane potential

**Figure 11:**
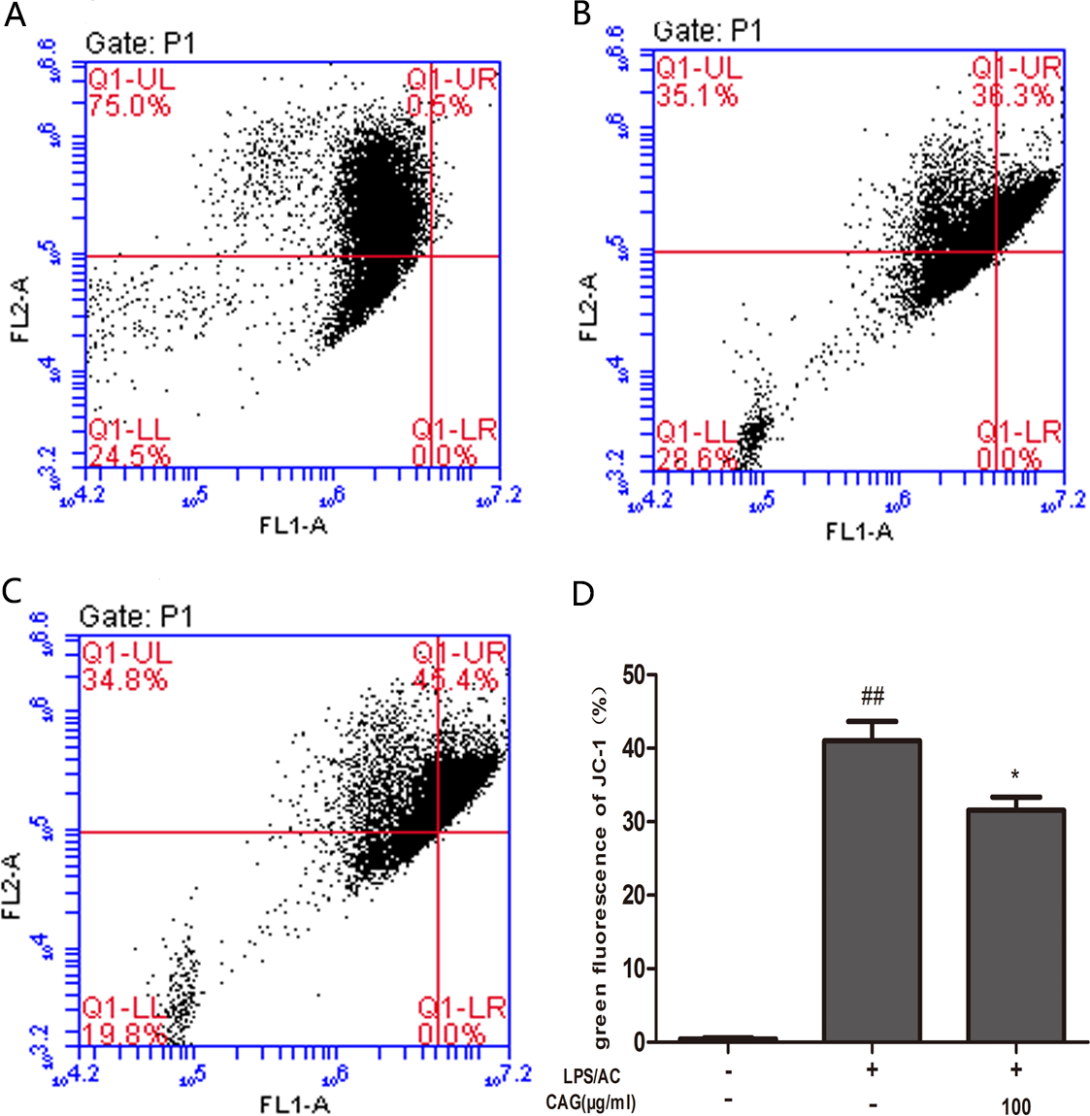
Effect of CAG on LPS/AC-induced changes in the mitochondrial membrane potential of chicken liver hepatocytes ΔΨm was measured in the (**A**) control group, (**B**) model group, (**C**) 100 μg/mL CAG treatment group. (**D**) Percentage of green fluorescence. The data are expressed as the mean ± SD (*n =* 3). ^##^ < 0.01 compared to the control group; * < 0.05 compared to the model group.

The ΔΨm (mitochondrial membrane potential) of chicken liver cells was analyzed using JC-1. Normal mitochondria initially fluoresced red, and then green as ΔΨm become lower. The treatment group showed increased green fluorescence compared with the control group. Furthermore, green fluorescence decreased upon treatment with CAG at 100 μg/mL (Figure 11).

## DISCUSSION

The objective of the present study was to explore the effect of LPS/AC in hepatocytes and to determine whether CAG could reverse such effect. We found that LPS/AC causes hepatocyte injury and that CAG can reverse it. In our experiments, cells treated with LPS/AC showed decreased viability, decreased ALT and AST activity, increase in the levels of oxidative stress indicators, increased apoptosis, and alterations in the expression levels of apoptosis-related mRNAs and proteins.

We used a modified two-step IV collagenase *ex-situ* perfusion method that was based on an *in situ* method to isolate primary hepatocytes as a predictor of liver responses [29, 30]. Our hepatocytes showed a high level of dispersion, purity, and viability (90%) in culture for approximately nine days (Figure 1). This allowed us to assess enzyme induction and inhibition and perform medium-throughput screening of compounds.

LPS and AC have each been shown to induce liver injury [18, 31]. A composite liver injury model using LPS and Bacillus Calmette-Guerin or D–galactosamine has been previously established [32–34]; however, no reports employing LPS in combination with AC to induce liver injury are currently available. In our previous study, we determined the optimal LPS and AC doses that induce liver injury *in vitro*, as well as their respectively IC_50_, which was 60 μg/mL and 180 μg/mL, respectively. On this basis, treatment of hepatocytes with LPS/AC resulted in a concentration-dependent increase in cell death, with an IC_50_ of 30 + 100 μg/mL, which indicates that the dose of LPS required to generate a combined AC induced liver injury was much lower than that required when these are modeled separately (Figure 2). Reports have shown that LPS can induce hepatocyte damage in rat primary hepatocytes at a dose of 40 μg/mL [35]. The dose we found to induce liver injury in chicken hepatocytes was thus higher than that in rats. This suggests that rat liver cells are more sensitive to LPS than chicken cells. However, when we exposed chicken hepatocytes to a combination of AC potassium and LPS, the concentration of LPS necessary to induce injury was lower than that in rats.

The present study reveals that the activity of ALT and AST in the cell culture medium of the model groups was higher than in controls (Figure 3). This suggests that LPS/AC causes hepatic structural damage since ALT and AST are normally localized in the cytoplasm and are released into the circulation only after cellular damage [36].

In our investigation, we treated hepatocytes with different doses of CAG after exposure to 30 μg/mL of LPS + 100 μg/mL of AC (Figure 3A). Results from our cell viability assays indicate that viability increased in a dose-dependent manner and was accompanied by an increase in the activity of ALT and AST in the supernatant (Figure 3). These results indicate that LPS/AC can induce liver injury *in vitro*, and that CAG can reverse this effect.

Oxidative stress can also activate signaling pathways, thereby causing cell damage [37, 38]. MDA, which is produced by free radical-mediated lid peroxidation, is frequently used as a marker of oxidative stress. In contrast, SOD, GSH, and GSH-Px protect host cells from oxidative damage by scavenging free radicals. Figure 4 shows an increase in MDA levels and a decrease in SOD activity, as well as a decrease in the concentration of GSH and GSH-Px in cell lysate collected from the model group. This suggests that LPS/AC triggers oxidative damage to the cell membranes of hepatocytes, in agreement with previous studies [39]. Furthermore, glycyrrhizin effectively inhibits lid peroxidation and enhances the capacity to eliminate free-radicals [28]. In addition, CAG inhibited LPS/AC-induced liver injury by increasing SOD activity and GSH levels and decreasing MDA levels (Figure 4), which may be attributable to its free-radical scavenging power. ROS can induce cell death and activate various signaling pathways [40]. Our study here we showed that CAG could decrease ROS induced by LPS/AC in hepatocytes.

Apoptosis in hepatocytes can occur in virus- or nonvirus-induced acute liver injury [41, 42] and is the main mechanism hallmark of liver failure [43]. To determine whether LPS/AC induces apoptosis in hepatocytes and whether CAG reverses this event, we measured the rate of apoptosis in cultured hepatocytes by FCM and Hoechst 33342 staining (Figures 5 and 6). Both experiments showed an increase in the number of apoptotic hepatocytes after LPS/AC treatment. However, when CAG was added into the medium, cellular repair was observed in a dose-dependent manner.

The inter-membranous space of mitochondria contains various pro-apoptotic proteins, which include cytochrome c, AIF, and endonuclease G (EndoG). Mitochondrial swelling and release of cytochrome occur during permeabilization of the outer mitochondrial membrane [44–46]. Cytochrome c associates with the adaptor protein Apaf-1 and the prodomain protein caspase-9 to form the apoptosome complex, which in turn initiates apoptosis [47, 48]. Caspase-3, which is a key executor of apoptosis, is then activated. Our results indicated that the mRNA and protein levels of caspase-3 increased after LPS/AC injection, whereas this was markedly attenuated by CAG (Figures 7 and 8). In our experiments, LPS/AC treatment also induced an increase in the mRNA and protein levels of caspase-9, cyt c, and bax, which in turn may be responsible for inducing apoptosis in primary hepatocytes. Furthermore, mitochondria became swollen and ΔΨm decreased in model group. Similarly, western blot analyses showed that cyt c was released from mitochondria into the cytoplasm (Figures 9, 10 and 11).

The bcl-2 family includes pro-apoptotic (bax and bid) or anti-apoptotic (bcl-2 and bcl-xl) proteins that regulate the mitochondrial apoptotic pathway. In addition, these proteins also regulate cell survival and death by blocking both the death receptor and mitochondrial apoptosis pathways [49]. Bcl-2 and bax have opposite effects on cell death: Bcl-2 inhibits or delays cell death, whereas bax accelerates apoptosis [50]. Our results here suggest that the increase in bax mRNA and protein levels and the decrease in Bcl-2 levels correlate with LPS/AC-induced apoptosis (Figures 7–8), in agreement with studies reporting that the bax/bcl-2 ratio increases with apoptosis [51]. The administration of 100 μg/mL of CAG attenuated bax mRNA and protein expression, suggesting that CAG has a protective effect against LPS/AC-induced liver injury.

**Figure 12:**
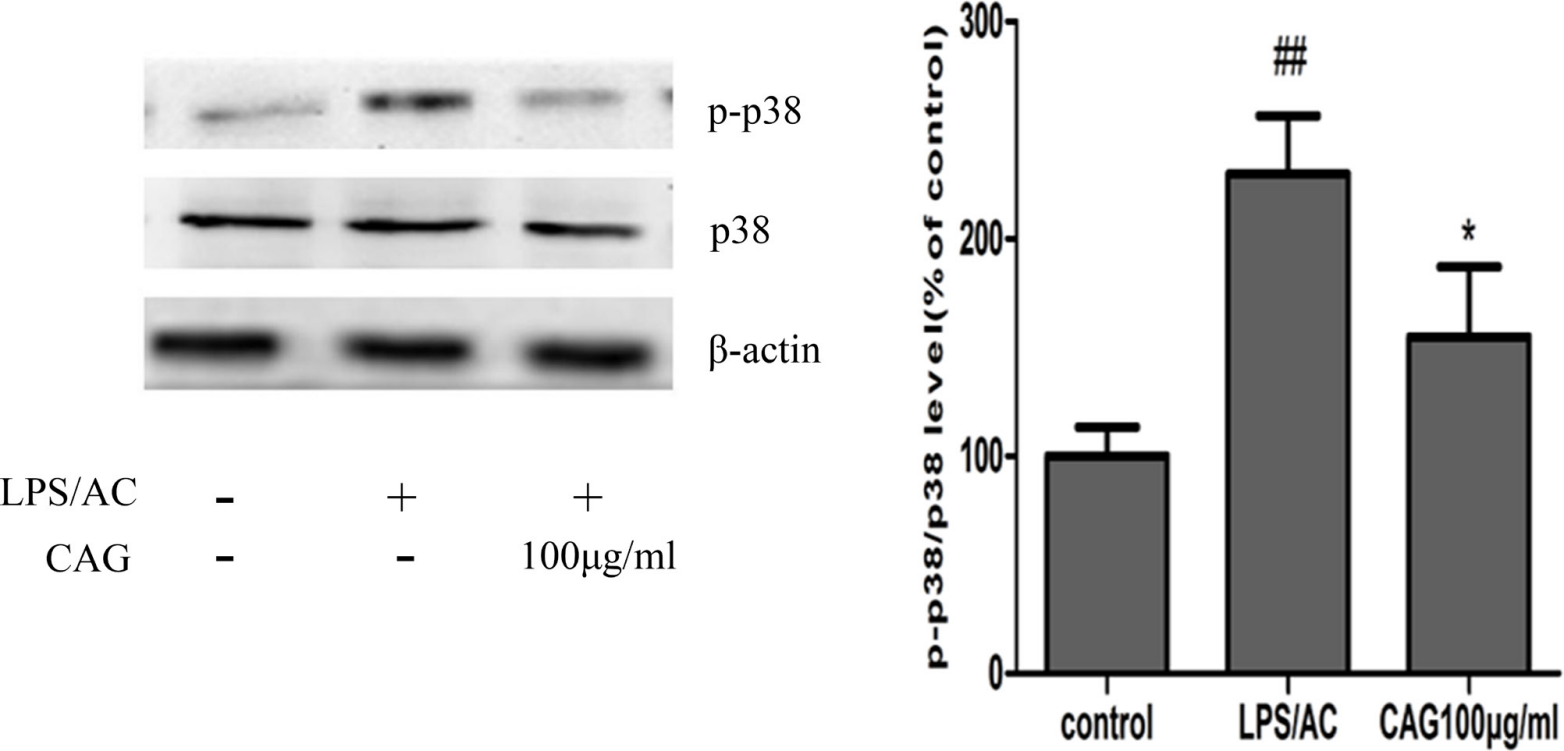
Effects of CAG treatment on p38 MAPK phosphorylation Three groups of cells were tested; namely, the control, the model and the 100 μg/mL CAG treatment groups. ^#^ < 0.05, ^##^ < 0.01 compared to the control group; * < 0.05, ** < 0.01 compared to the model group.

The p38 MAPK pathway transduces a variety of extracellular signals that transmit cellular responses to stress, and is implicated in cell proliferation, differentiation, and apoptosis [52, 53]. In the present study, we observed an increase in the rate of phosphorylation of p38 kinases after exposure to LPS/AC, an effect that was reversed after CAG treatment (Figure 12). The hepatoprotective activity of CAG may thus be due to the inhibition of LPS/AC-induced phosphorylation of p38 MAPKs.

In conclusion, this is the first model that depicts LPS/AC-induced chicken liver injury *in vitro* and the beneficial effects of CAG treatment. The mechanism of action of CAG as a protective agent involves the maintenance of the level of cellular antioxidants, inhibition of apoptosis, and regulation of the p38 MAPK signaling cascade in liver cells. Therefore, our study shows that AC potassium enhances the toxicity of LPS in chicken hepatocytes whereas CAG administration may be used an alternative therapy to treat or prevent acute hepatic damage.

## MATERIALS AND METHODS

### Reagents and materials

CAG was prepared in our laboratory. Collagenase (type IV), LPS (*E. coli* L-2880) and HPEPS were purchased from Sigma Chemical Co. Dulbecco’s modified eagle’s medium (DMEM) was obtained from Hyclone. Diagnostic kits used to determine the presence or absence of AST, ALT, SOD, GSH, and MDA were obtained from Nanjing Jiancheng Institute of Biotechnology (Nanjing, China).

### Preparation and incubation of isolated hepatocytes

Hepatocytes were isolated from male Hailan chickens (average weight: 1.0–1.5 kg) by using a two-step collagenase perfusion method. The chickens were anesthetized by using a cocktail of xylazine (2 mg/mL) and ketamine (20 mg/mL), and their livers were excised after ligating the hepatic blood vessels such as the pancreaticoduodenal veins, the mesenteric vein, and the inferior caval vein. We then cut a ventage in the portal vein and inserted a tubule to allow perfusion of saline solution A (33 mM/L HEPES, 127.8 mM/L NaCl, 3.15 mM/L KCl, 0.7 mM/L Na_2_HPO_4_·12H_2_O, 0.6 mM/L EGTA, pH 7.4) that was warmed to 37°C to wash out the blood. Then, to remove the EGTA, perfusion was performed using saline solution B (solution A added 3 mM/L CaCl_2_, pH 7.4) at room temperature and at a flow rate of 10–20 mL/min for 15 min. Finally, the liver was perfused in a beaker with 0.5% collagenase IV at a flow of 20 mL/min for 20–25 min at 37°C. Hepatocytes were separated from other cellular components by centrifugation at 50g for 3 min and the precipitates were resuspended in DMEM containing 10% fetal bovine serum (FBS, Gibco), 0.5 mg/L bovine insulin, 100 U/mL penicillin, and 0.25 mg/mL streptomycin (pH 7.4). Hepatocytes were counted using a hemocytometer, and cell viability was determined using Trypan blue. Cells were resuspended in DMEM and diluted to a final concentration of 5 × 10^*5*^ cells/mL. The hepatocytes were seeded onto plates and then incubated at 37°C in a humidified incubator with an atmosphere of 5% CO_2_.

### Establishment of a LPS/AC-induced liver injury model

Cells were seeded onto 96-well plates at a density of 5 × 10^*5*^ cells/mL in 200 μL of incubation culture medium for 24 h. Then, the hepatocytes were incubated with LPS/AC at concentrations of 30 + 60, 30 + 80, 30 + 100, 30 + 120, and 30 + 140 μg/mL for 24 h. MTT stock solution (5 mg/mL) was then applied to each of the wells, and the cells were incubated in a humidified atmosphere for 4 h. The absorbance of the samples was measured using a microtiter plate reader at a dual wavelength mode of 490 nm and 655 nm. Cell viability was calculated using the following equation: Cell viability = (Average OD of the treated wells - Average OD of the blank wells)/ (Average OD of the control wells - Average OD of the blank wells) × 100%, where OD is the optical density.

### Evaluation of the protective effect of CAG on the LPS/AC-induced hepatocyte injury

Hepatocytes were seeded onto 96-well plates at the same density for 24 h. Different batches of cells were then incubated with CAG at concentrations of 1, 10, and 100 μg/mL for 24 h. After incubation, the supernatant was discarded, and the cells were exposed to LPS/AC for 24 h at a concentration that induces death of 50% of the hepatocytes. Cell viability was measured as described above (Methods 2.3). The levels of AST and ALT in the cell culture supernatant were measured using commercial kits according to the manufacturer’s protocols.

### Determination of MDA levels and SOD, GSH, and GSH-Px

The cells were washed twice with 300 μL of phosphate buffered saline (PBS, pH 7.4). The cells were detached using a sterilized scraper and lysed in 25 mmol/L Tris-HCl lysis buffer. The homogenate was then sonicated on ice (10-s pulses) and finally centrifuged at 13,000 g for 15 min. The supernatant was collected and analyzed according to the procedures recommended by the manufacturers of the respective assay kits.

### ROS detection

ROS levels were assessed using DCFH-DA. Briefly, after the cell medium was discarded, the cells were incubated with DCFH-DA for 20 min at 37°C, washed three times with PBS, and then visualized under a fluorescence microplate.

### Measurement of caspase-3 activity

The cells were isolated using trypsin, centrifuged (4°C, 2,000 rpm, 5 min), and resuspended in 150 μL of a lysis buffer and 1.5 μL DTT from a caspase-3 colorimetric assay kit (KGA202, KeyGEN, China), placed on ice for 1 h, and vortexed four times for 10 s each time. The cell suspension was centrifuged (10,000 rpm, 1 min), and the supernatant was transferred to a 1.5-mL centrifuge tube, which was then placed on ice. Approximately 50 μL of a 2× reaction buffer and 5 μL of the caspase-3 substrate were added to each 50-μL sample, incubated at 37°C in the dark for 4 h, and then analyzed using a spectrophotometer at a wavelength of 405 nm.

### Determination of apoptosis in primary chicken liver cells by flow cytometry (FCM)

The detection kit (eBioscience, USA) employed in this study utilizes FITC-conjugated annexin V/PI. After treating with LPS/AC and CAG, the hepatocytes were dissociated by using 0.25% trypsin without EDTA (Solarbio, China) and collected as a suspension. A 100-µL aliquot of the cell suspension was transferred to a 5-mL culture tube, which was then mixed with 5 μL of Annexin V-FITC and 5 μL of propidium iodide. The cells were gently vortexed and incubated for 15 min at room temperature (25°C) in the dark. Then, 400 µL of 1× binding buffer were added to each tube and analyzed by FCM within 1 h.

### Detection of apoptosis via Hoechst 33342 staining

Primary chicken liver cells were cultured on a glass slide in 24-well plates. The cells were washed with PBS and then stained with Hoechst 33342 (1 mg/mL, Nanjing Jiancheng, China) for 10 min. After two washes with PBS, the cells were examined using fluorescence microscopy.

### Relative quantification of target gene expression by real-time PCR (RT-PCR)

Total RNA was extracted using TRIzol*™* (9108/9109 Takara, Japan) according to the manufacturer’s instructions. cDNA synthesis was performed using 1 μg of total RNA using a cDNA synthesis kit (RR047A Takara, Japan). The mRNA expression levels of caspase-3, bax, bcl-2, Fas, and actin were quantified by RT-PCR using a SYBR green premix according to the manufacturer’s instructions (RR820A Takara, Japan). The thermal cycling conditions of the PCR assays were as follows: denaturation at 95°C for 3 min, followed by 40 cycles of denaturation at 95°C for 5 s, primer annealing at 58°C for 30 s, and primer extension at 72°C for 1 min, with a final extension at 72°C for 6 min. For each PCR product, a single narrow peak was obtained by melting curve analysis at a specific temperature. The relative expressions of the target genes were normalized to that of actin. The data were calculated using the 2^*-ΔΔCt*^ method as described by the manufacturer and were expressed as a fold-increase over the indicated control groups.

### Relative quantification of target protein expression by western blot

Hepatocytes were homogenized in RIPA buffer containing 50 mM Tris/HCl (pH 8), 150 mM NaCl, 1% non-idet-P40, 1% sodium deoxycholate, 0.1% SDS, 0.1 mM DTT, 0.05 mM PMSF, 0.002 mg/mL aprotinin, 0.002 mg/mL leupeptin, and 1 mM NaVO_3_. After centrifugation at 10,000 g for 20 min, the protein concentration of the upper layer was determined by using a BCA kit according to the manufacturer’s instructions. Approximately 50 μg of the lysate was then resolved by SDS-PAGE and transferred to nitrocellulose membranes (Immobilon, Millipore Corp, Bedford, MA, USA). The membrane was blocked with TBST buffer (50 mM Tris-HCl, 150 mM NaCl, 0.05% (w/v) Tween-20, pH 7.5) containing 5% dried non-fat milk, then reacted with polyclonal caspase-3 (1: 1,000, Abcam, ab115183, UK), caspase-9 (Abcam, ab115161, UK), bax (EnoGene, E1A0083-1, China), Bcl-2 (BD, 610538, USA), and cyt c (1:100, Santa Cruz, sc-13560, USA) overnight at 4°C. The membranes were washed with TBST thrice, each for 10 min, and then incubated with a horseradish peroxidase-labeled antibody (1: 1,000 dilution) for 40 min at room temperature. After incubation, the membranes were thoroughly washed with TBST, and immunoreactive bands were detected by using ECL reagents.

### Transmission electron microscopy (TEM)

For electron microscopy, the cells of every group earlier described were rapidly fixed in 2.5% glutaraldehyde in 0.1 M sodium phosphate buffer (pH 7.2) for 3 h at 4°C, and then later used in preparing TEM sections. Ultrathin sections were stained with 2% uranyl acetate and observed under a Hitachi transmission electron microscope.

### Mitochondrial membrane potential assay

JC-1 kit was used to measure the mitochondrial membrane potential of the liver cells. After treatment with LPS/AC and CAG, cells were trypsinized, collected, centrifuged, resuspended with 0.5 mL DMEM and 0.5mL JC-1 stain liquid, and incubated for 20 min. Cells were washed two times with JC-1 stain buffer solution, then analyzed with FCM.

### Statistical analysis

All data were expressed as the mean ± standard deviation. One-way ANOVA was used for statistical comparisons. A *p* value of < 0.05 was deemed statistically significant. Graphs were plotted using Graphpad Prism 4 (GraphPAD Software, San Diego, CA, USA).
